# ΔCT Value of Amplified Refractory Mutation System Predicts Efficacy of EGFR-TKIs in Advanced Non–Small-Cell Lung Cancer: A Multi-Center Retrospective Study

**DOI:** 10.3389/fmolb.2021.684661

**Published:** 2021-10-08

**Authors:** Min Chen, Wenqi Huang, Dongyong Yang, Jincheng Huang, Gong Li, Xiaoqing Wang, Nanjie Xiao, Weijian Zhang, Jian Guan, Shuang Wang, Laiyu Liu

**Affiliations:** ^1^ Department of Radiation Oncology, Nanfang Hospital, Southern Medical University, Guangzhou, China; ^2^ Department of Radiation Oncology, Peking University Shenzhen Hospital, Shenzhen, China; ^3^ Chronic Airways Diseases Laboratory, Department of Respiratory and Critical Care Medicine, Nanfang Hospital, Southern Medical University, Guangzhou, China; ^4^ Department of Respiratory and Critical Care Medicine, Second Affiliated Hospital of Fujian Medical University, Quanzhou, China; ^5^ Guangdong Provincial Hospital of Traditional Chinese Medicine, Guangzhou, China; ^6^ Department of Radiation Oncology, The First Affiliation Hospital of Fujian Medical University, Fuzhou, China; ^7^ Department of Pathology, Nanfang Hospital, Southern Medical University, Guangzhou, China; ^8^ Department of Pathology, School of Basic Medical Sciences, Southern Medical University, Guangzhou, China

**Keywords:** ΔCT, ARMS, efficacy, EGFR-TKIs, NSCLC

## Abstract

**Purpose:** This multi-center retrospective study determines whether the ΔCT value of the Amplified Refractory Mutation System (ARMS) predicts the efficacy of epidermal growth factor receptor (EGFR) tyrosine kinase inhibitors (TKIs) in EGFR-mutant advanced non–small-cell lung cancer (NSCLC).

**Patients and methods:** Patients who harbored an exon 19 deletion (19Del) or L858R mutation detected by the ARMS and previously received treatment of EGFR-TKIs as a monotherapy were enrolled. A total of 108 NSCLC patients in four hospitals were enrolled. We divided the patients into a high ΔCT group (Group H) and a low ΔCT group (Group L) by the Martingale residuals analysis and log-rank test. The primary outcome was progression-free survival (PFS). Univariate analysis and multivariable regression were applied to compare the PFS between the groups.

**Result:** The Martingale residuals analysis and log-rank test were applied to find the cutoff ΔCT value (0.8). In the 108 patients we enrolled, 59 were in group L and 49 were in group H. Patients’ demographics and clinical characteristics, including age, sex, smoking history, pathology, mutation sites, TNM stage, and line of TKIs therapy, were not significantly different between group L and group H. The median PFS was 11.1 months in group L and 6.9 months in group H, and the difference showed statistical significance (*p* < 0.001). Moreover, the objective response rates (ORRs) in group L was significantly higher than in group H (61.0 vs 34.7%, *p* = 0.002). The median OS was 25.0 months in group L and 20.0 months in group H (*p* = 0.046).

**Conclusion:** The ΔCT value of ARMS could be an efficacy predictor for EGFR-TKI treatment in advanced EGFR-mutant NSCLC.

## Introduction

Lung cancer is the most common cancer leading to cancer-related deaths worldwide. More than 70% of patients with lung cancer are diagnosed with advanced non–small-cell lung cancer (NSCLC) (Siegel et al., 2017). Many clinical trials have demonstrated the superiority of epidermal growth factor receptor (EGFR) tyrosine kinase inhibitors (TKIs) over chemotherapy in the treatment of patients with advanced NSCLC harboring EGFR mutations (Maemondo et al., 2010; Mitsudomi et al., 2010; Rosell et al., 2012; Yang et al., 2013; Wu et al., 2014). Therefore, EGFR-TKIs have been recommended as the standard of care for first-line treatment for EGFR-mutant NSCLC, especially for those who harbored a drug sensitivity-associated mutation including exon 19 deletion (19Del) and exon 21 L858R. However, these clinical trials also showed that the efficacy of EGFR-TKIs was not so satisfactory in a nonnegligible proportion of NSCLCs harboring sensitive EGFR mutations. In the studies we have mentioned above (Maemondo et al., 2010; Mitsudomi et al., 2010; Rosell et al., 2012; Yang et al., 2013; Wu et al., 2014), 20–44% patients who harbored EGFR mutation had a best response of stable disease (SD) or progressive disease (PD) and 8–15% patients had a best response of PD. Still a significant minority of patients had a primary resistance or poor progression of disease (PFS) to EGFR-TKIs when harbored sensitive EGFR mutations.

Distinguishing patients who are most likely to experience an expected response to EGFR-TKIs from those who are not likely to show a response has emerged as a crucial issue. Although previous studies had reported several resistance mechanisms of EGFR TKIs for EGFR-mutant NSCLC patients, in many cases, the mechanisms remain unclear (Costa et al., 2014; Beau-Faller et al., 2014; Ogino et al., 2007; Engelman et al., 2007). Efforts are still needed to explore the reasons for the various rates of resistance to EGFR-TKIs for EGFR-mutant NSCLC.

The correlation of intratumor heterogeneity of tumor and drug resistance has been widely studied in recent years (Turner and Reis-Filho, 2012; Jamal-Hanjani et al., 2015; Mangano et al., 2015). For the EGFR heterogeneity in lung cancer, recent reports have indicated that tumors are composed of mixed populations of mutant EGFR and wild-type EGFR cells, suggesting that the intratumor heterogeneity does indeed exist (Jiang et al., 2008; Taniguchi et al., 2008; Wei et al., 2014; Cai et al., 2015). Furthermore, several groups have demonstrated that the intratumor heterogeneity of EGFR-mutant NSCLC associated with the efficacy of EGFR-TKIs. They demonstrated that patients with higher relative abundance of EGFR mutation showed longer PFS (Jiang et al., 2008; Taniguchi et al., 2008; Zhou et al., 2011; Zhao et al., 2014a; Zhao et al., 2014b). These reports gave a great inspiration to the development of the therapeutic strategy of EGFR-TKIs. Researchers advised that for patients who harbored a high ratio of EGFR mutation in tumor, EGFR-TKI was effective to control the progression of tumor, but for the low, monotherapy of EGFR-TKIs may be not enough. However, until now, there was not any effective and convenient ways for clinicians to distinguish whether a patient is harboring a high ratio of EGFR mutation in tumor or not. Therefore, there is an urgent need to develop a method to assess the abundance of *EGFR* mutations in NSCLC in clinical practice. The Amplified Refractory Mutation System (ARMS) had widely been applied in the detection of EGFR mutation in recent years (Shaozhang et al., 2014). Mutant allele assays were run with a gene reference assay, which was designed to a mutation-free region of the gene. The mutational status of a sample was determined by calculating the ΔCT value between amplification reactions for a mutant allele assay and gene reference assay, as follows: ΔCT = Ct (mutant allele assay) – Ct (gene reference assay). Mutation or not is determined by the Ct value and ΔCT value. The reference gene was a relatively conserved region of the EGFR gene. Thus, Ct (gene reference assay) could effectively reflect the DNA level of the EGFR gene. For the tumor samples with EGFR mutation, the ΔCT value could reflect the relative level of EGFR mutation in the tumor sample. Therefore, we assume that patients with a lower ΔCT value may harbor a high ratio of EGFR mutation and may associate with better response to EGFR-TKIs. Furthermore, we divided the EGFR-mutant NSCLC into a low ΔCT value and a high ΔCT value to explore whether the ΔCT value of the tumor sample could be a predictor for the efficacy of EGFR-TKIs in EGFR-mutant NSCLC patients.

### Patients and Methods

#### Patients

A total of 108 Chinese patients were enrolled in this study from four medical centers in China including Nanfang Hospital of Southern Medical University, the first affiliated hospital of Fujian Medical University, the second affiliated hospital of Fujian Medical University, and Guangdong Province Traditional Medical Hospital between March 2013 and Jan 2015. The criteria for the patients enrolled in this retrospective study were as follows: 1. Diagnosed with advanced NSCLC and harbored a drug sensitivity-associated mutation site (19Del and L858R). 2. EGFR mutations were tested by ARMS (Shanghai Yuanqi Bio-Pharmaceutical Company Limited, Shanghai, China) and previously received treatment of EGFR-TKIs including gefitinib, erlotinib and icotinib as a monotherapy. The data we collected of all patients were from the electronic medical record system in the four medical centers.

### Epidermal Growth Factor Receptor Mutation Analysis

EGFR mutation testing was performed on formalin-fixed paraffin-embedded (FFPE) specimens from primary tumor obtained from bronchoscopic biopsy or CT-guided core biopsy before any tumor-related treatment. The HE-stained section of FFPE was assessed again to establish the pathological diagnosis. Some of the FFPE samples were trimmed according to the HE-stained section to make sure that the samples we would test were all tumors. The genomic DNA was isolated and purified from tumor specimens using the DNeasy Blood & Tissue Kit (Qiagen, Valencia, CA, United States) according to the manufacturer’s instructions. EGFR mutation detection was performed according to the principles of the ARMS, using the Human EGFR Gene Mutation Detection Kit (PCR Fluorescence Probe) (Shanghai Yuanqi Bio-Pharmaceutical Company Limited, Shanghai, China) on the MX3005P QPCR system (Agilent, Santa Clara, CA, United States), according to manufacturers’ recommendations. It covered 23 EGFR mutation hotspots within exons 18, 19, 20 and 21. The results were analyzed according to the criteria defined by the manufacturer’s instructions. Positive results were defined as Ct (mutant allele assay) – Ct (gene reference assay) < ΔCT (cutoff). We divided the patients into two groups by the media ΔCT value.

### Statistical Analyses

The primary end point was progression-free survival (PFS), and the second end points were the objective response rate (ORR) and primary resistance. In patients with measurable disease, the tumor burden was assessed by the Response Evaluation Criteria in Solid Tumor (RECIST) and categorized as a complete response (CR), partial response (PR), stable disease (SD), or progressive disease (PD) (Eisenhauer et al., 2009). PFS was calculated from the time from commencement of EGFR-TKIs treatment to PD according to the RECIST criteria or death resulting from any cause. OS was calculated from the time from commencement of EGFR-TKI treatment to death. The definition of primary resistance to EGFR-TKIs was not uniform among researchers, and in this study, we defined it as patients who had progressive disease to EGFR TKI without initial objective response (Cortot and Janne, 2014). The Kaplan–Meier method was applied to analyze the PFS or OS, and the Cox proportional hazard model was applied to explore the statistical difference in PFS between different groups. A comparison of ORRs and rates of primary resistance in different groups was made using χ2 tests. A two-sided *p* value of less than 0.05 was considered statistically significant.

## Result

### Patient Characteristics

A total of 108 patients who fully met the enrollment criteria were enrolled in the present study. In all the patients, the median age was 61 (ranged from 40 to 83 years); 52 were males and 56 were females; 63 were never smokers and 45 were current or former smokers; three were non-adenocarcinoma and 105 were adenocarcinoma; 57 patients harbored a 19Del mutation and 51 harbored a L858R mutation; and 87 patients received EGFR-TKI as the first-line therapy, 20 patients received EGFR-TKI as the second line therapy, and 1 received EGFR-TKI as the third-line therapy.

### Epidermal Growth Factor Receptor Mutation Groups

**FIGURE 1:**
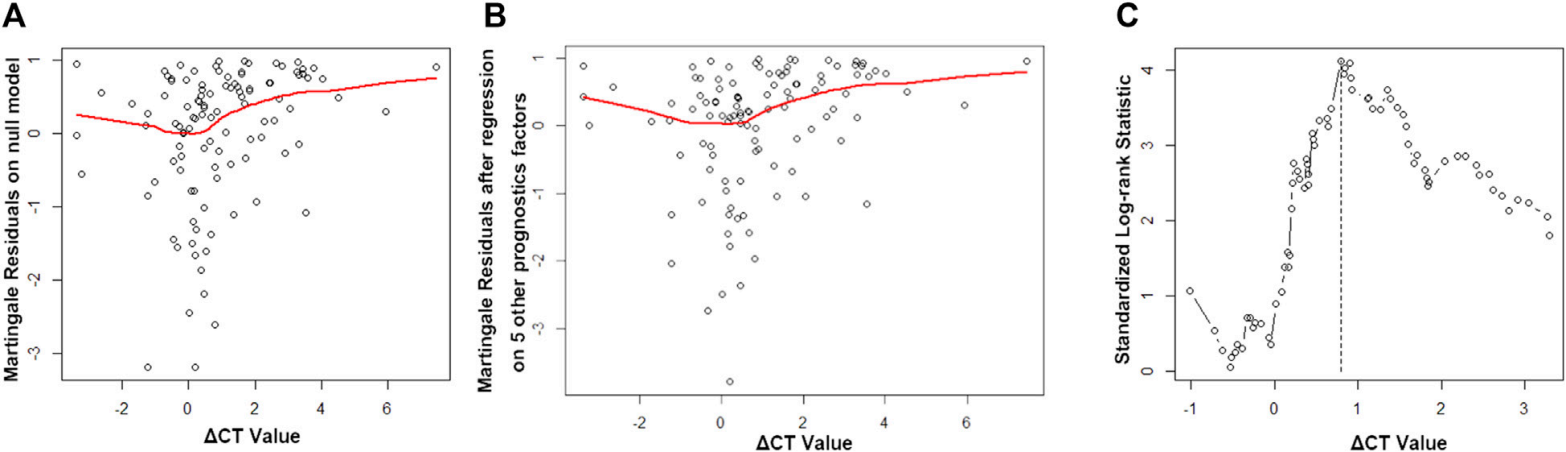
Martingale residuals analysis and standard log-rank analysis. **(A)** Martingale residuals analysis of the association between the ΔCT value and progression-free survival (PFS). **(B)** Martingale residuals analysis of the association between the ΔCT value and PFS by age, gender, smoking status, mutation sites, and therapy line (first-line or second-line therapy). **(C)** Standard log-rank statistic to determine the cutoff value.

Martingale residuals analysis was applied, and a non-linear relationship was noted between the ΔCT value and PFS of the patients (Figures 1A,B). The risk of event went down with an increasing ΔCT value when ΔCT < −1, remained steadily when −1 > ΔCT <1, and increased when ΔCT >1. Thus, we applied log-ranking analysis to find the cutoff ΔCT value. The ΔCT value corresponding to the maximum log-rank value was defined as the cutoff ΔCT value and was 0.8, which agreed with the tendency in the Martingale residuals analysis (Figure 1C).

59 patients had a ΔCT value less than 0.8 (group L), and 49 patients had a ΔCT value greater than 0.8 (group H). The median of age was similar between group L and group H (60 vs 61, *p* = 0.499). 49.2% of group L and 55.1% of group H were female (*p* = 0.538). The proportions of never smokers in group L and group H were similar (71.2 vs 71.4%, *p* = 0.978), and 19Del was more frequent in group L than L858R mutation (61.0 vs 42.9%, *p* = 0.060). The TNM stage and line of TKI therapy were all well balanced between the two groups (*p* = 0.356, *p* = 0.818). Treatment with different EGFR-TKIs was balanced between the two groups (*p* = 0.422) (Table 1).

**TABLE 1:** Patient demographics and clinical characteristics.


		ΔCT value					
		Group L (n = 59)			Group H (n = 49)		
Variable	Total	No. of patients	%		No. of patients	%	P
Age, years							0.499
Median (range)	61 (40–83)	60 (40–83)		61 (41–79)			
Sex							0.538
Male	52	30	50.8		22	44.9	
Female	56	29	49.2		27	55.1	
Smoking history							0.978
Never-smoker	77	42	71.2		35	71.4	
Current or former-smoker	31	17	28.8		14	28.6	
Pathology							0.412
Non-adeno	3	2	3.4		1	1.9	
Adeno	105	57	96.6		48	98.1	
Mutation site							0.060
19Del	57	36	61.0		21	42.9	
L858R	51	23	39.0		28	57.1	
TNM stage							0.356
IIIB	7	5	8.5		2	4.1	
IV	101	54	91.5		47	95.9	
Line of therapy							0.818
First	87	48	81.4		39	79.6	
Second	21	11	18.6		10	20.4	
EGFR-TKIs							0.422
Gefitinib	50	26	44.1		24	49.0	
Erlotinib	44	27	45.8		17	34.7	
Icotinib	14	6	10.2		8	16.3	

Group L: patients with a lower ΔCT value; group H: patients with a higher ΔCT value; adeno: adenocarcinoma.

### Efficacy of Different ΔCT Value Groups

All the patients were received TKIs from Jan 2013 to Dec 2015 in the four hospitals. The last follow-up date was Aug 9, 2021.100 (92.6%) patients experienced a disease progression, and 36 (33.3%) of the patients were still alive or concord.

**FIGURE 2:**
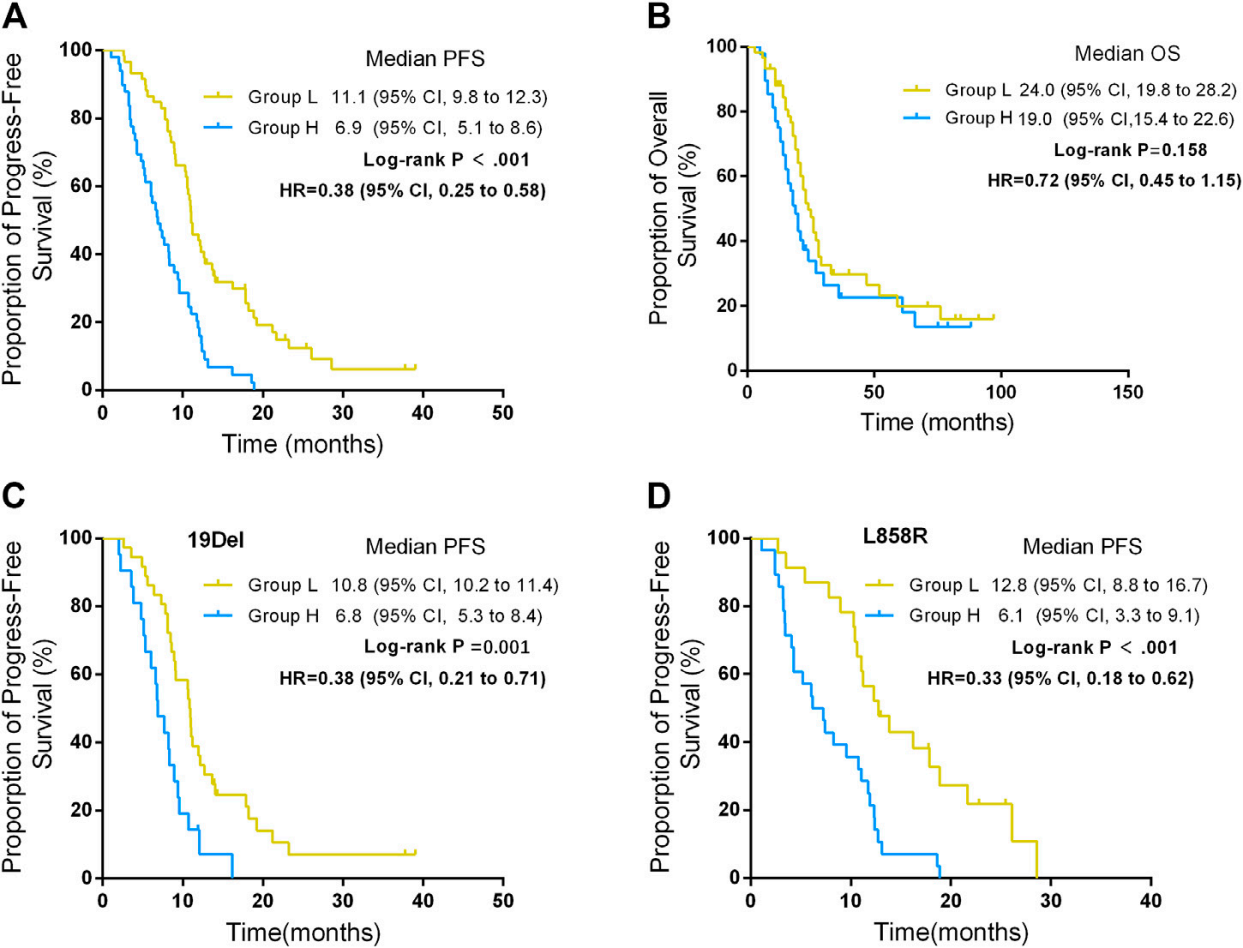
Progression-free survival (PFS) and overall survival (OS) between different ΔCT value groups. **(A)** PFS between different ΔCT value groups. **(B)** OS between different ΔCT value groups. **(C)** PFS between different ΔCT value groups in patients harboring *EGFR* 19Del. **(D)** PFS between different ΔCT value groups in patients harboring *EGFR* L858R.

The median PFS was 11.1 months (95% CI, 9.8–12.3) in group L and 6.9 months (95% CI, 5.1–8.6) in group H, and the difference showed statistical significance (*p* < 0.001) (Figure 2A). Multivariate analysis shows that the ΔCT value was the variable that mostly influences the PFS (Table 2).

**TABLE 2:** Multivariate analyses of PFS by Cox regression analysis.

Variable	HR	95% CI	*p* Value
**ΔCT value groups**			
Group L	0.358	0.231–0.555	<0.001
Group H		Reference	
**Mutation site**			
19Del	1.299	0.848–1.989	0.229
L858R	Reference		
**Gender**			
Male	1.055	0.612–1.820	0.847
Female	Reference		
**Age**	0.980	0.956	1.004
**Smoking or not**			
Never-smoker	1.311	0.727–2.363	0.368
Current or former-smoker	Reference		
**Line of therapy**			
First	1.311	0.727–2.363	0.096
Second	Reference		

PFS, progression free survival; HR, hazard ratio; CI, confidence interval.

The rate of primary resistance in group H was significantly higher than in group L (26.5% *vs* 5.1%, *p* = 0.002). ORRs in group L were significantly higher than in group H (61.0% *vs* 34.7%, *p* = 0.002). The median OS was 24.0 months (95% CI, 19.8–28.2) in group L and 19.0 months in group H (95% CI, 15.4–22.6), which was statistically significant (*p* = 0.046). (Table 3; Figure 2B).

**TABLE 3:** Efficacy of different ΔCT value groups.


		ΔCT value					
		Group L (*n* = 59)		Group H (*n* = 49)			
Variable	Total	No. of patients	%	No. of patients		%	P
PFS, days							<0.001
Median		11.1 9.8 to 12.3		6.9 5.1 to 8.6		
95% CI						
OS, days							0.158
Median		24.0		19.0			
95% CI		19.8–28.2		15.4–22.6			
Primary resistance							0.002
Yes	16	3	5.6	13		24.1
No	92	56	94.9	36		73.5	
Tumor response							0.002
CR	0	0	0	0		0	
PR	53	36	61.0	17		34.7	
SD	39	20	33.9	19		38.8	
PD	16	3	5.1	13		26.5	

PFS, progression free survival; OS, overall survival; CR, complete response; PR, partial response; SD, stable disease; PD, progressive disease; CI, confidence interval.

In addition, for patients who either harbored 19Del or L858R mutation, patients in group L had better PFS than those in group H. For patients who harbored 19Del mutation, the median PFS was 10.8 months (95% CI, 10.2–11.4) in group L and 6.8 months (95% CI, 5.3–8.4) in group H, and the difference was statistically significant (*p* = 0.001) (Figure 2C). For patients who harbored L858R mutation, the median PFS was 12.8 months (95% CI, 8.8–16.7) in group L and 6.1 months (95% CI, 3.3–9.1) in group H, and the difference showed statistical significance (*p* < 0.001) (Figure 2D).

### Efficacy of EGFR 19Del and EGFR 21 L858R Mutation

The PFS of patients who harbored 19Del mutation was 9.1 months (95% CI, 7.9–10.2), and in patients who harbored L858R mutation, PFS was 10.6 months (95% CI, 8.5–12.7). The difference between the two group was not significant (*p* = 0.634) (Figure 2A). ORRs in patients who harbored 19Del were higher than in those who harbored L858R mutation, while the difference showed no significance (59.7 vs 39.2%, *p* = 0.034). However, the rate of primary resistance was significantly higher in patients with L858R mutation than those with 19Del (23.5 vs 6.3%, *p* = 0.018) (Table 4).

**TABLE 4:** Efficacy of EGFR 19Del and EGFR 21 L858R mutation.


		Mutation sites					
		E19Del (n = 57)		L858R (n = 51)			
Variable	Total	No. of patients	%	No. of patients		%	P
PFS, months							0.643
Median		9.1		10.6			
95% CI		7.9–10.2		8.5–12.7			
OS, months							
Median		25.3		21.6			0.241
95% CI		21.0–29.7		18.0–25.2			
Primary resistance							0.018
Yes	16	4	6.3		12	23.5	
No	92	53	93.7		39	76.5	
Tumor response							0.026
CR	0	0	0		0	0	
PR	54	34	59.7		20	39.2	
SD	38	19	33.3		19	37.3	
PD	16	4	7.0		12	23.5	

PFS, progress free survival; OS, overall survival; CR, complete response; PR, partial response; SD, stable disease; PD, progressive disease; CI, confidence interval.

**FIGURE 3:**
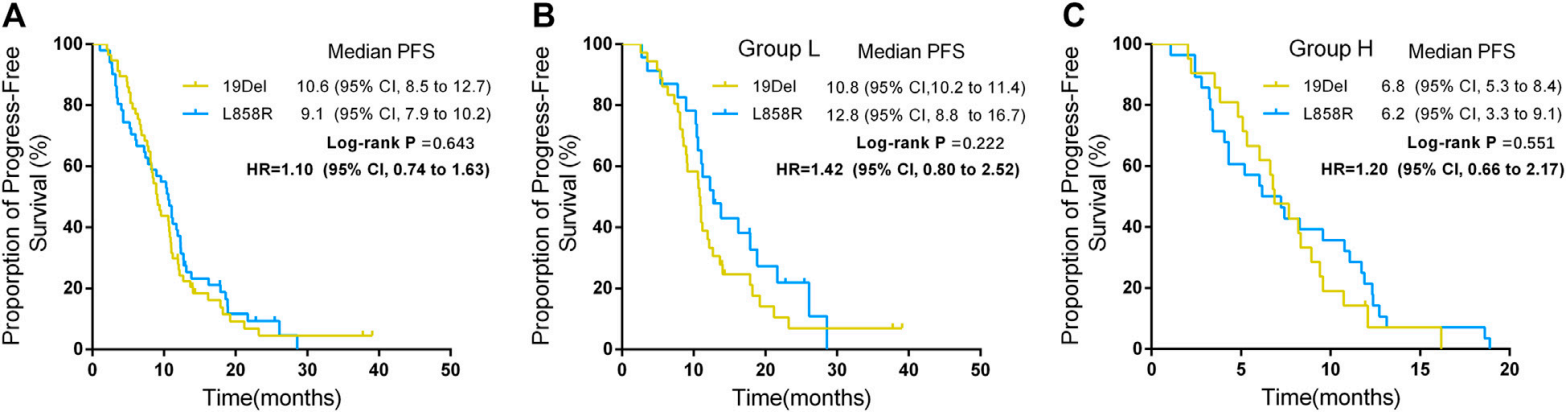
Progression free survival (PFS) between different mutation sites and groups. **(A)** PFS between different mutation sites. **(B)** PFS between different mutation sites in group L. **(C)** PFS between different mutation sites in group H.

In addition, for patients in group L, the PFS of the patients who harbored 19Del was 10.8 months (95% CI, 10.2–11.4) and the PFS of the patients who harbored L858R was 12.8 months (95% CI, 8.8–16.7) (*p* = 0.222) (Figure 3B). For patients in group H, the PFS of the patients who harbored 19Del mutation was 6.8 months (95% CI, 5.3–8.4) and the PFS of the patients who harbored L858R was 6.2 months (95% CI, 3.3–9.1) (*p* = 0.551) (Figure 3C).

## Discussion

Our study put forward a new possible predictor for efficacy of EGFR-TKIs. We first put forward that the ΔCT value from the ARMS–PCR in EGFR mutation testing could be a predictor for efficacy of EGFR-TKI treatment for advanced EGFR-mutant NSCLC. Patients with a lower ΔCT value of EGFR mutations may benefit more than those with a higher ΔCT value of EGFR mutations according to the statistically different PFS between the groups, and PFS of the two groups had no overlaps. This might partly explain why some patients who harbored sensitive EGFR mutation did not experience an expected duration of response to EGFR-TKIs. Just like the previous studies, the data of the present study made us not only to focus on whether the patients harbored an EGFR mutation but also to consider the relative abundance of EGFR mutations when making therapeutic strategies for NSCLC (Jiang et al., 2008; Taniguchi et al., 2008; Zhou et al., 2011; Zhao et al., 2014a; Zhao et al., 2014b). What is further than the previous research was that we put forward a predictor that could be conveniently applied in clinical practice. By relatively quantifying the EGFR mutations in tumor tissue according to ΔCT value, patients with a low ΔCT value of EGFR mutations could receive EGFR-TKI treatment because they would benefit the most. However, for patients with a high ΔCT value of EGFR mutations, monotherapy of EGFR-TKIs may not be enough to control the tumor progression. Owing to the fact that 45.4% of the patients were still alive or concord, overall survival was required further to follow up.

Sheng et al. (2015) conducted a meta-analysis including 26 studies and showed that patients with NSCLC and EGFR exon 19 deletion had a longer PFS, OS, and higher ORR compared with exon 21 L858R mutation after EGFR-TKI therapy. Zhang et al. (2017) thought they may be two distinct diseases. However, it remains unclear why the difference in the outcome exists between the two mutation sites. In our study, ignoring the absence of statistical significance, exon 19Del was more frequent in the group of the low ΔCT value and showed higher rate of ORR and lower rate of primary resistance to EGFR-TKIs than exon 21 L858R. Although the PFS showed similarity in the two groups, it may be a clue for researchers to find out the reason why exon 19Del has a better response to EGFR-TKIs. For us, studies with large samples will be conducted to further our findings.

There were some advantages and disadvantages in the present study. The first advantage is that this was a multi-center retrospective study and all the data we recorded were from the electronic medical record system, which made our result more reliable. The second advantage is that the predictor we found in this study was much more applicable in clinical practice than in other previous studies.

The first limitation of this study is that the patients in our study had a tumor response evaluation per 8–12 weeks after EGFR-TKIs were taken. However, the proportion of patients had an evaluation per 8–10 weeks and per 11–12 weeks, which were not significantly different. The second limitation is that the follow-up of OS was not regular in our study and might lead to a large bias. However, our aim of this study was to explore whether the ΔCT value affects the efficacy of EGFR-TKIs. The third one was that only one site of the tumor sample was obtained from each patient. Nonetheless, we trimmed some of the tumor sample to ensure that every sample content is a cancerous cell to make our results more reliable. Wei et al. (2014) have demonstrated that the EGFR mutation ratio showed a high level of concordance in primary tumors and when the tumor content is more than 50% in a tumor sample, a randomly chosen sample would reliably represent the type and ratio of mutations of EGFR in primary tumors.

## Conclusion

In summary, our study suggests that the ΔCT value of EGFR mutations could predict the efficacy of EGFR-TKI treatment in EGFR mutant NSCLC. We hope this indicator would contribute to more accurate technologies to evaluate the ratio of EGFR mutation in tumors.

## Data Availability

The original contributions presented in the study are included in the article/Supplementary Material; further inquiries can be directed to the corresponding authors.
